# Pediatric sleep-disordered breathing in Shanghai: characteristics, independent risk factors and its association with malocclusion

**DOI:** 10.1186/s12903-023-02810-9

**Published:** 2023-03-08

**Authors:** Yuanyuan Li, Xianqin Tong, Shuai Wang, Liming Yu, Gang Yang, Jinqiu Feng, Yuehua Liu

**Affiliations:** 1 Department of Pediatric Dentistry, Shanghai Stomatological Hospital & School of Stomatology, Fudan University, Shanghai, China; 2 Shanghai Key Laboratory of Craniomaxillofacial Development and Diseases, Fudan University, Shanghai, China; 3 Department of Orthodontics, Shanghai Stomatological Hospital & School of Stomatology, Fudan University, Shanghai, China

**Keywords:** Sleep-disordered breathing, Prevalence, Risk factor, Malocclusion, Cross-sectional study

## Abstract

**Objectives:**

This study aimed to determine the prevalence and independent risk factors of SDB, and explore its association with malocclusion among 6–11-year-old children in Shanghai, China.

**Methods:**

A cluster sampling procedure was adopted in this cross-sectional study. Pediatric Sleep Questionnaire (PSQ) was applied to evaluate the presence of SDB. Questionnaires including PSQ, medical history, family history, and daily habits/environment were completed by parents under instruction, and oral examinations were implemented by well-trained orthodontists. Multivariable logistic regression was applied to identify independent risk factors for SDB. Chi-square tests and Spearman's Rank Correlation were used to estimate the relationship between SDB and malocclusion.

**Results:**

A total of 3433 subjects (1788 males and 1645 females) were included in the study. The SDB prevalence was about 17.7%. Allergic rhinitis (OR 1.39, 95% CI 1.09–1.79), adenotonsillar hypertrophy (OR 2.39, 95% CI 1.82–3.19), paternal snoring (OR 1.97, 95% CI 1.53–2.53), and maternal snoring (OR 1.35, 95% CI 1.05–1.73) were independent risk factors for SDB. The SDB prevalence was higher in children with retrusive mandibles than in proper or excessive ones. No significant difference was observed in the correlation between SDB and lateral facial profile, mandible plane angle, constricted dental arch form, the severity of anterior overjet and overbite, degree of crowding and spacing, and the presence of crossbite and open bite.

**Conclusions:**

The prevalence of SDB in primary students in the Chinese urban population was high and highly associated with mandible retrusion. The independent risk factors included Allergic rhinitis, adenotonsillar hypertrophy, paternal snoring, and maternal snoring. More efforts should be made to enhance public education about SDB and related dental-maxillofacial abnormalities.

## Introduction

Sleep-disordered breathing (SDB) is a common syndrome characterized by upper airway (UA) dysfunction during sleep, and subsequent sleep disruption and ventilation abnormalities. It includes a range of clinical entities of varying severity from primary snoring to obstructive sleep apnea/hypopnea syndrome (OSAHS) in children of all ages [1]. Epidemiological studies in different age groups and regions showed that the prevalence of SDB varied greatly, ranging from 7 to 27.6% [2–8].

Adenoid vegetations and/or adenotonsillar hypertrophy, and craniofacial abnormalities associated with decreased UA volume are considered the main predisposing factors for SDB [1, 9–12]. Other risk factors such as obesity and neuromuscular disorders have been identified [11, 13]. There are also studies indicating that SDB has been associated with several controversial risk factors, including a history of prematurity, allergic diseases, asthma, tobacco exposure, and parental history of adenotonsillectomy [1, 3, 7, 14–16]. Several studies suggested that breastfeeding may be a protective factor against SDB in childhood [12, 17, 18]. while other studies did not support these results. Such discrepancies in potential risk/protective factors may be due to ethnicity and age differences among study populations and how SDB is defined.

SDB in children is commonly correlated with craniofacial dentofacial features and may be related to dental-maxillofacial abnormalities [9, 19–21]. It was reported that patients with SDB often experience alterations in the tongue’s position, and posture of the head and neck, which may break the balance of oral and peri-oral muscles, consequently posing a negative impact on the development of the craniofacial skeleton and dental occlusion according to Moss’s theory [22]. Lyra et al. [23] conducted a cross-sectional work of 390 children and found that overjet, anterior open bite, and posterior crossbite were significantly associated with SDB. Two earlier cross-sectional studies reported a correlation between SDB and open bite or convex facial profile respectively [24, 25]. Although children with SDB and malocclusion may share some anatomical features, the relationship between SDB and malocclusion in children is still controversial.

The incidence of atopic diseases and adenotonsillar hypertrophy, and the living conditions of children in China are changing rapidly. Meanwhile, the prevalence of SDB may be changing as well. The prevalence of dentofacial deformity among the Chinese population of children and adolescents, as reported [26, 27], has increased by 27% since the 1960s. With people’s emerging attention to mouth breathing, the orthodontic consultation rate caused by mouth breathing has hugely increased in China. Quite a few clinicians suggested that children with SDB should be routinely examined by orthodontists. However, SDB and malocclusion are still issues rarely mentioned by parents in well-child visits and by primary health care providers. This study aimed to assess the prevalence and risk factors of SDB and its relation to malocclusion in primary school children in Shanghai, China. This study enrolled 3433 children of 6–11 years and included an assessment of SDB risk factors and potentially related malocclusion derived from previous reports and our clinic experience.

## Study design and participants

Shanghai Stomatological Hospital conducted an epidemiological survey of the oral health status of primary school students in Shanghai. A cluster sampling procedure was adopted in this cross-sectional study. Four primary schools were randomly selected in the suburban district (Minhang district) and two in the urban district (Jingan district). The students in grades one to five were identified as potential subjects for the survey. The exclusive criteria were as follows: tooth agenesis, orofacial clefts, and other congenital malformation; children unable to cooperate; whose guardians do not agree to join the study.

This study was approved by the Ethical Committee of the Shanghai Stomatological Hospital on 24 December 2021 (Certificate Number 2021–028) and started after that day. Informed consent was obtained from the guardians of subjects before the survey.

## Procedure

The research was mainly comprised of questionnaires completed by parents under the instruction and examinations implemented by orthodontists.

### Questionnaire

The first part of the questionnaire was about general information on subjects such as sex, birthday, height, and weight. The second part was Pediatric Sleep Questionnaires (PSQ), which were applied to evaluate the possible presence of SDB. The PSQ includes questions about the frequency and severity of snoring and apnea, mouth breathing, daytime sleepiness, the problem of attention or behaviour, and other pediatric symptoms of SDB. The average score of all items greater than 0.33 was defined as the presence of SDB [28]. The third part of the questionnaire contained 8 questions about medical history and family history. The Last part was about daily habits/environment for SDB, such as tobacco smoke exposure (more than 20 min a day recorded as yes), sleeping position (supine, lateral, or prone), frequency of using air conditioner, air humidifier, and air cleaner (more than 30 days/year recorded as commonly used) in the house.

### Examination

The examinations were implemented by five orthodontists with more than 3 years of clinical experience. Before the examination, the orthodontists had accomplished standardized training. The inter-examiner reliability was insured by Cohen’s kappa coefficient (value > 0.8). The examinations were performed in schools’ infirmaries using portable lighting and disposable mouth mirrors. Each examiner was assigned a recorder to fill in the paper version of the health check form simultaneously. 13 items were included in the form.

1. Neck circumference: measured in the middle of the neck using tape.
2. Lateral facial profile: concave, upright, or protruding. It was assessed by the aesthetic plane (from nasal tip to pogonion of soft tissue) while the subject was sitting in a comfortable upright position.
3. Mandible development: excessive, proper, or retrusive. The position of the upper lip was used as a reference.
4. Sagittal relationship of molar occlusion: Angle class I, II or III. The classic Angle classification was applied.
5. Mandible plane angle: low, average, or high. It was assessed by the Frankfort plane.
6. Upper dental arch: normal or constricted maxillary dental arch.
7. Lower dental arch: normal or constricted mandible dental arch.
8. Overjet of anterior teeth: 0–3 mm: normal; 3–5 mm: mild; 5–8 mm: moderate; > 8 mm: severe. The distance from the palatal surface of the most protruded maxillary incisor to the labial surface of the corresponding mandibular incisor.
9. Crossbite or edge-to-edge: present or absent. It was considered present if the maxillary incisor occluded lingually to or onto the corresponding mandibular incisor.
10. Overbite of anterior teeth: ≤ 1/3, normal; 1/3–1/2, mild; 1/2–2/3, moderate; > 2/3, severe. It was assessed by the coverage of the mandibular incisors by most of the maxillary incisors.
11. Open bite: present or absent. It was considered present if the overlap of the lower incisors by upper incisors in the vertical plane was less than 0 mm.
12. Teeth crowding: 0 mm: normal; 0–4 mm: mild; 4–8 mm: moderate; > 8 mm: severe.
13. Teeth spacing: 0 mm: normal; 0–2 mm: mild; 2–4 mm moderate; > 4 mm: severe.

## Statistical analysis

The paper version of the health check form of subjects was input to Epidata software by two data processors from Shanghai KNOWLANDS MedPharm Consulting Co. Ltd. One inspector was responsible for quality control.

Data were analyzed using SPSS Statistics 23 (SPSS Inc., Chicago, IL, USA) software package, adopting the both-sided significance level of 5%. Continuous variables were expressed as mean ± standard deviation. The Student’s t-test was used for continuous variables including height, weight, BMI, and neck circumference. We assessed the significance of differences by the Chi-square test for categorical variables. Based on Chi-square tests, variables with a *p* value ≤ 0.05 were selected for the pool of potential risk factors in building multivariate models. Multivariable logistic regression was applied to identify risk factors for SDB. The relationship between the prevalence of SDB and dental-maxillofacial development was evaluated using the Chi-square tests and Spearman correlation analyses.

## Results

Among 4230 students from the six schools, 3853 families agreed to participate in the survey, 3499 returned the questionnaire, and 3457 children completed the oral examination. After excluding data with obvious errors, a total of 3433 subjects (1788 males and 1645 females, mean age of 8.53 ± 1.47 years) were contained in the study.

The characteristics of subjects and distribution of children with SDB were summarized in Tables 1 and 2. The prevalence of SDB among primary school children defined by PSQ was about 17.7%. Moreover, it was much higher in boys (21.0%) than in girls (14.0%) (*P* < 0.001). No difference was observed between age groups (*p* > 0.05) (Table 2). Greater body weight (*P* < 0.05) and BMI (*P* < 0.001) can be observed in children with SDB. Neck circumference was longer in SDB children, but no significant difference was observed (*p* > 0.05) (Table 1).

**Table 1:** Basic information of non-SDB and SDB children

	Total (n = 3433)	Male (n = 1788)	Female (n = 1645)	*P* (male vs. female)	Non-SDB (n = 2827)	SDB (n = 606)	*P* (N-SDB vs. SDB)
Height (cm)	134.7 ± 10.3	135.0 ± 9.8	134.3 ± 10.8	< 0.05	134.8 ± 10.3	134.3 ± 9.9	0.37
Weight (kg)	32.0 ± 9.0	32.8 ± 8.9	31.2 ± 8.9	< 0.001	31.9 ± 8.9	32.9 ± 9.5	< 0.05
BMI	17.5 ± 3.9	17.8 ± 3.8	17.1 ± 4.0	< 0.001	17.3 ± 3.8	18.0 ± 4.5	< 0.001
NC (cm)	28.1 ± 2.6	28.6 ± 2.7	28.1 ± 2.6	< 0.001	28.1 ± 2.6	28.3 ± 2.7	0.06

*BMI* body mass index, *NC* neck circumference

The Student’s t-tests were used

**Table 2:** The prevalence of SDB in different age groups and sex groups

	Total (n)	Non-SDB	SDB	Prevalence of SDB (%)	χ2	*P*
*Age (years)*						
6–7	685	576	109	15.9	9.36	0.053
7–8	775	655	120	15.5		
8–9	822	662	160	19.5		
9–10	546	433	113	20.7		
10–11	605	501	104	17.2		
*Sex*						
male	1788	1413	375	21.0	28.31	< 0.001
female	1645	1414	231	14.0		
Total	3433	2827	606	17.7	–	–

Chi-squared tests were used

The potential risk factors for SDB were described in Table 3. Allergic rhinitis and adenotonsillar hypertrophy are well-known risk factors for SDB in children. In this study, the prevalence of SDB in children with medical histories of allergic rhinitis, adenotonsillar hypertrophy, or asthma or tympanitis was much higher than that in children without these medical histories (*P* < 0.001). As shown in Table 3, parents’ rhinitis history or parents’ snoring were associated with SDB (*P* < 0.001). As for daily habits, tobacco exposure and sleeping position were associated with the prevalence of SDB. Children exposed to tobacco for more than 20 min a day had a higher prevalence of SDB (*P* < 0.01). The prevalence of SDB was significantly higher in children who usually slept in prone positions than in supine or lateral positions (*P* < 0.001). The frequency of using an air conditioner, air humidifier, and air cleaner in the house did not correlate with SDB (*p* > 0.05).

**Table 3:** Univariate analysis of potential risk factors for SDB in children

		Total	Non-SDB	SDB	Prevalence (%)	χ2	*P* value
*Medical history*							
Allergic rhinitis	Yes	1309	993	316	24.1	68.9	< 0.001
	No	1999	1740	259	13.0		
Adenotonsillar hypertrophy	Yes	391	260	131	33.5	99.3	< 0.001
	No	2748	2372	376	13.7		
Asthma	Yes	164	119	45	27.4	11.17	< 0.001
	No	3231	2683	548	17.0		
Tympanitis	Yes	306	228	78	25.5	14.2	< 0.001
	No	3127	2599	528	16.9		
*Family history*							
Paternal rhinitis history	Yes	1055	837	218	20.7	10.81	< 0.001
	No	2315	1944	371	16.0		
Maternal rhinitis history	Yes	789	621	168	21.3	10.43	< 0.001
	No	2600	2176	424	16.3		
Paternal snoring	Yes	2127	1672	455	21.4	58.79	< 0.001
	No	1157	1033	124	10.7		
Maternal snoring	Yes	634	478	156	24.6	27.61	< 0.001
	No	2613	2201	412	15.8		
*Daily habits/environments*							
Tobacco exposure	Yes	836	663	173	20.7	7.31	< 0.01
	No	2579	2151	428	16.6		
Sleeping position	Supine	1156	956	200	17.3	14.16	< 0.001
	Lateral	1970	1642	328	16.7		
	Prone	307	229	78	25.4		
Air conditioner	Commonly	401	332	69	17.2	0.062	0.8
	Rarely	3032	2495	537	17.7		
Air humidifier	Commonly	584	485	99	17.0	0.237	0.63
	Rarely	2849	2342	507	17.8		
Air cleaner	Commonly	906	758	148	16.3	1.47	0.23
	Rarely	2527	2069	458	18.1		

Chi-square tests were used

**Fig. 1:**
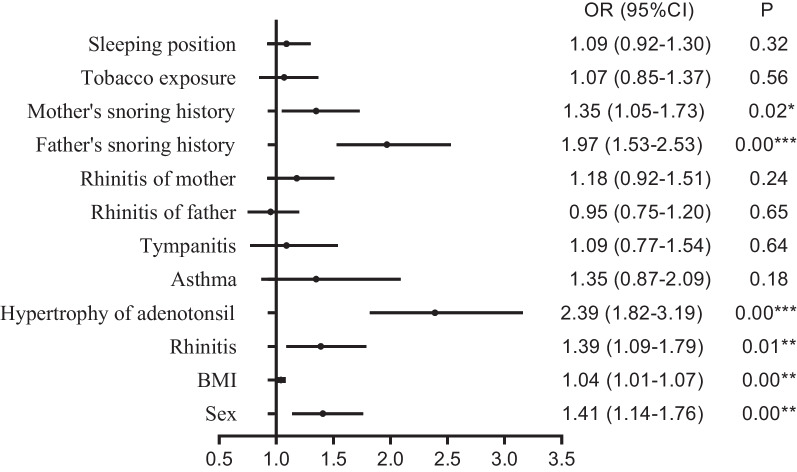
Multivariate regression analysis of risk factors for SDB

In Fig. 1, multivariate logistic regression showed that allergic rhinitis (OR 1.39, 95% CI 1.09–1.79), adenotonsillar hypertrophy (OR 2.39, 95% CI 1.82–3.19), paternal snoring (OR 1.97, 95% CI 1.53–2.53), and maternal snoring (OR 1.35, 95% CI 1.05–1.73) were independent risk factors of SDB. However, the medical histories of asthma or tympanitis, parents’ rhinitis history or tobacco exposure or sleeping position, and SDB did not demonstrate significant association by multivariate regression analysis.

Among the dental-maxillofacial variables, only mandible development was significantly associated with SDB (Table 4). Children with SDB were more susceptible to retrusion of the mandible (χ2 = 6.82, *P* < 0.05). Children with protruding profiles intended to have a higher frequency of SDB than those in upright/concave profiles, although it did not show a statistical difference (*p* > 0.05). Children with molar occlusion of Angle class I showed a non-significantly lower SDB prevalence than Angle class II and III occlusion. Spearman analyses of the correlation between SDB and mandible plane angle, constricted dental arch form, the severity of anterior overjet and overbite, degree of crowding and spacing, and the presence of crossbite and open bite were conducted, but no significant correlation was observed in this study (Table 5).

**Table 4:** The association between SDB and dental-maxillofacial growth

	Total	Non-SDB	SDB	Prevalence of SDB (%)	χ2	*P* value
*Lateral facial profile*						
Concave	128 (3.8%)	107	21	16.4	1.43	0.49
Upright	2103 (61.8%)	1744	359	17.1		
Protruding	1174 (34.4%)	955	219	18.7		
*Mandible development*						
Excessive	52 (1.6%)	44	8	15.4	6.82	0.03*
Proper	2409 (73.8%)	2001	408	16.9		
Retrusive	802 (24.6%)	634	168	20.9		
*Molar occlusion*						
Class I	2064 (63.4%)	1718	346	16.8	4.44	0.11
Class II	996 (30.6%)	799	197	19.8		
Class III	198 (6.0%)	160	38	19.2		
*Mandible plane angle*						
Low	203 (6.3%)	163	40	19.7	0.71	0.70
Average	2462 (76.3%)	2031	431	17.5		
High	560 (17.4%)	458	102	18.2		

Chi-square tests were used

* Statistically significant at *P* < 0.05

**Table 5:** Spearman correlation analysis of SDB and malocclusions

	Rho	*P* value
Upper dental arch	− 0.01	0.56
Lower dental arch	0.00	0.86
Anterior overjet	0.02	0.31
Anterior crossbite	0.48	0.49
Anterior overbite	− 0.01	0.60
Anterior open bite	0.41	0.52
Maxillary crowding	0.02	0.33
Mandibular crowding	− 0.01	0.79
Maxillary spacing	0.01	0.49
Mandibular spacing	0.00	0.84

*Rho* Spearman's Rank Correlation Coefficient

## Discussion

SDB can cause various complications, such as abnormal growth and cardiovascular, immunological, and metabolic disorders, affecting a child’s health and quality of life for many years [29]. Currently, SDB-related dentofacial abnormalities draw more attention from parents and doctors in communities than that decades ago. We call for more efforts from local health authorities to enhance public propaganda and education on prevention measurements for SDB and related diseases. Meanwhile, more attention should be paid to further studies on dental-maxillofacial growth.

The golden standard method for SDB diagnosis is polysomnography (PSG). However, there are restrictions on applying PSG in large-scale population-based epidemiological surveys and health screenings [30]. Therefore, the PSQ, a tool validated to have a sensitivity of 85% and a specificity of 87% for identifying children with SDB, was used in this study [28]. Li et al. [31] confirmed the applicability and generalizability of the Chinese version of PSQ in an extensive epidemiological survey of pediatric SDB in a cross-sectional study in China.

A total of 3433 subjects (1788 males and 1645 females) aged 6–11 years from primary school in Shanghai were included in this study. The overall prevalence of SDB was 17.7%. It was reported that the prevalence of pediatric SDB ranged from 8.5 to 11.3% in a large cohort of primary schools in Japan [32]. In another study of a random sample of 3–11-year-old children in Wuxi, China, SDB prevalence was about 13.4% [2]. The prevalence of SDB in this survey was higher than previously reported, which may be attributed to the discrepancy in population subgroups, age-range, and environments.

The study showed that SDB was much more common in boys (21.0%) than in girls (14.0%). This was consistent with many previous study reports [3, 33, 34]. Children with SDB weighed about 1 kg more than children without SDB (non-SDB) and averaged about 0.7 points higher in BMI. Although some studies did not observe the association between SDB and weight [23], it is almost accepted that obesity plays an important role in the pathophysiology of pediatric SDB [11, 35, 36].

Different from adults, the major risk factor of pediatric OSAHS is currently reported as adeno-tonsillar hypertrophy [37, 38]. Adenotonsillar hypertrophy directly leads to the narrowing of the retropalatal part of the upper airway, which is usually the most common site of obstruction due to the smallest cross-sectional. The most frequent cases of a high incidence of SDB caused by adenotonsillar hypertrophy were found in 3–5 years old children [39]. It is generally accepted that the number of respiratory infections decreased in 6–11-year-old children, and their SDB is usually associated more closely with allergic disease [4, 40, 41]. Allergic rhinitis may influence sleeping by several mechanisms, and the main point is that the increased airway resistance is caused by nasal congestion due to the nasal mucosa allergic inflammatory. Using PSG and a questionnaire, Liu et al. studied allergic rhinitis’s influence on SDB. They found that despite the high prevalence of allergic rhinitis in children with SDB, allergic rhinitis was only linked to behavioral problems rather than the aggravating factor for SDB when assessed by PSG [42]. In this study, we found a high frequency of SDB with rhinitis (24.1% vs. 13.0%), adenotonsillar hypertrophy (33.5% vs. 13.7%), asthma (27.4% vs. 17.0%) and tympanitis (25.5% vs. 16.9%). The multivariate logistic regression showed that allergic rhinitis (OR 1.39) and adenotonsillar hypertrophy (OR 2.39) were risk factors for SDB, while asthma and tympanitis were not. Some studies identified asthma as a vital factor for SDB [2, 43]. However, we did not obtain a positive result, possibly due to the low proportion of children with asthma and the inability to distinguish between rhinitis and asthma.

Parental snoring has long been identified as a significant risk factor for pediatric SDB in different ethnic groups [44, 45]. Kannan et al. [46] explored the predictors of childhood habitual snoring in a birth cohort and found that parental habitual snoring was consistently associated with childhood habitual snoring from ages 1 to 7. Our study supported these observations and proposed that paternal snoring (OR 1.97) and maternal snoring (OR 1.35) were risk factors for SDB.

We also evaluated the relationship between daily habits/environment and SDB prevalence. We did not observe an association between the frequency of using air conditioners, air humidifiers, and air cleaners and the SDB prevalence. It was speculated that tobacco exposure and air pollution might be linked to an increased risk of respiratory diseases such as allergies, airway inflammation, and oedema, resulting in an increased prevalence of SDB. In our study, children exposed to tobacco for more than 20 min a day had a higher prevalence of SDB (20.7% vs. 16.6%). However, the differences were not significant in multivariate logistic regression analysis. As for sleep position, the prevalence of SDB was much higher in children who usually slept in the prone position (25.4%) than in the supine (17.3%) or lateral position (16.7%). Sleep in the prone position is more commonly seen in children than adults, and it differs from the supine position incontrolling the cardiovascular, respiratory, and thermoregulatory systems. Numerous clinicians believe that sleeping in a supine position is more likely to induce the collapse of the upper airway and lead to SDB. However, this conjecture was proved to be a fault in this study and previous research which was also conducted in China [2], although the differences were insignificantin multivariate logistic regression analysis.

Dentofacial development is a complex biological process that could be affected and remodeled by various risk factors breaking the balance. The prevalence of dentofacial deformity among the Chinese population of children and adolescents, as reported [26, 27], has increased by 27% since the 1960s. SDB harms the healthy development of children but may also be associated with malocclusion due to some craniofacial anatomical characteristics. Our cross-sectional research explored if SDB is associated with malocclusion in 6–11-year-old children in China. We discovered that children with protruding profiles intended to have a higher frequency of SDB than upright/concave profiles, although it did not show a statistical difference. Children with molar occlusion of Angle class I showed a non-significantly lower SDB prevalence than Angle class II and III occlusion. Furthermore, the incidence of SDB in children with mandible retraction was significantly higher.

A follow-up study examined 6–8-year-old children from Finland at baseline and 2.2 years later and reported that children with SDB were more likelyhave the convex facial profile and mandibular retrusion than children without SDB [47], which was in agreement with previous works [25, 48]. However, the association between SDB and pediatric dentofacial deformity remained controversial. Lyra et al. [23] suggested that the prevalence of SDB was highly correlated with posterior crossbite and anterior open bite, but not with molar relationship or anterior overjet/overbite. It was reported that the dimensions of UA are smaller in children with large overjet and constricted dental arch, which might predispose them to SDB [49, 50], However,our study did not detect an association between overjet and dental arch. In a systematic review, Hansen et al. 2022 checked out the 1996 literature, and four papers were included last. They hold that no exact association can be ascertained between specific malocclusion and SDB in children [51]. We speculate that craniofacial morphology and dental occlusion seem to have complex, multifactor pathogenesis in children. Further research is needed to ascertain if SDB is associated with some specific malocclusion traits. Although the results of our study cannot confirm a definite risk of SDB to malocclusion but found a high prevalence of SDB in children with retrusive mandible, we suggested regular comprehensive epidemiological research of malocclusion and dental-maxillofacial healthy development guidance was needful for primary students.

The main limitation of this report was the definition of SDB. Due to the epidemiological nature of the study, the sample size was too large to obtain PSG data. A questionnaire completed by the guardian was used to evaluate the presence of SDB. Although PSQ is a widely used methods to assess SDB, some guardians may have needed to be more explicit about their children’s sleeping patterns and filled out PSQ inaccurately. Secondly, information regarding medical history reported by parents were not verified via thorough medical evaluation and documentation in medical records. Furthermore, some variables selected as a qualitative representative of malocclusion, the lateral profile, for example, might not satisfy a more detailed quantified analysis in the present study. We encourage that complementary examinations such as radiography and cephalometry be used to determine the malocclusion traits in further studies.

In conclusion, allergic rhinitis, adenotonsillar hypertrophy, paternal snoring, and maternal snoring were independent risk factors for pediatric SDB. In the study population, the prevalence of SDB was associated with retrusive mandible. We encourage patients with a history of allergic rhinitis, adenotonsillar hypertrophy, and paternal/maternal snoring, in addition to having mandibular retrusion should be referred for medical diagnosis of SDB, such as PSG monitoring, in order to prevent and minimize adverse effects of SDB on individuals lives. More efforts from local health authorities should be made to enhance public education on preventing and interrupting SDB and related dental-maxillofacial abnormalities.

## Data Availability

The datasets generated and analyzed during this study are included in this published article and its supplementary information files.
